# Longitudinal metagenomics reveals continuous restructuring of soil pathobiome under persistent *Phytophthora* pressure

**DOI:** 10.3389/fpls.2025.1749879

**Published:** 2026-02-02

**Authors:** Umer Basu, Shafat Ahmad Ahanger, Xiaotong Gai, Xiaoping Hu

**Affiliations:** 1 Yunnan Academy of Tobacco Agricultural Sciences, Kunming, China; 2 State Key Laboratory of Crop Stress Resistance and High-Efficiency Production, Key Laboratory of Plant Protection Resources and Pest Management of Ministry of Education, Key Laboratory of Integrated Pest Management on the Loess Plateau of Ministry of Agriculture and Rural Affairs, College of Plant Protection, Northwest A&F University, Yangling, Shaanxi, China

**Keywords:** diversity, KEGG orthologs, metagenome, microbiome, pathobiome, *Phytophthora nicotianae*, resistome, virulence

## Abstract

Soil borne pathogen, *Phytophthora nicotianae* causes black shank disease in tobacco, present a pervasive threat to global agriculture, with conventional control strategies often proving inadequate. A critical gap exists in our understanding of the long-term, dynamic interplay between the pathogen and the soil microbiome. To address this, we conducted a six-year longitudinal metagenomic study in a monocultured tobacco field, revealing a pathobiome in constant, non-equilibrium adaptation. Our analysis uncovered profound microbial restructuring, beginning with cumulative transcriptional reprogramming of highly significant genes. Functional profiling showed a critical metabolic shift toward anabolic capacity, with a 66.7% increase in KEGG orthologs and enrichment of amino acid biosynthesis (+8.9%), ribosomes (+13.0%), and quorum sensing (+11.0%). The soil resistome underwent dramatic succession, featuring an initial coordinated defense (R²=0.825), a comprehensive collapse in Year 3-4 (917 downregulated genes), and a resilient recovery that drove a net increase in antibiotic resistance, indicating a lasting ecosystem alteration. Virulence factor evolution revealed strategic trade-offs, with flagella systems dominating (2,583 occurrences) while more costly energy consuming secretion systems declined, and 87 core virulence factors persisted across time. Crucially, we observed a widespread decoupling between genetic potential and functional expression; key categories for defense and signal transduction declined in abundance (slopes of -150.4 and -264.9, respectively) despite stable gene counts, suggesting a systemic, energy conserving survival strategy. Concurrently, the community experienced progressive diversity loss (Shannon index slope = -0.0464/yr at genus level) despite maintained species richness (717 species), indicating restructuring was driven by shifting evenness rather than species loss. Our findings exhibit that persistent pathogen pressure drives the soil microbiome into a continuous state of adaptive restructuring, prioritizing coordinated defensiveness and metabolic efficiency over stability. This time resolved framework challenges static views of soil ecosystems and provides a foundational dataset for developing predictive, microbiome informed strategies to manage soil borne diseases sustainably.

## 1 Introduction

For Microbes form complex, active networks with plants that are crucial for plant health and ecosystem function. These interactions can boost plant growth, nutrient uptake, and stress resistance through processes like nitrogen fixation and antibiotic production. However, they can also be harmful, with the balance between good and bad microbes determining plant health (Berendsen et al., 2012; Trivedi et al., 2020). Soil borne pathogens are a major threat to global food production. This group includes destructive fungi, oomycetes, and bacteria, that cause diseases like root rot and wilts, leading to severe crop losses (Berendsen et al., 2012; Dubey et al., 2019). They are difficult to control because they can survive for years in the soil as tough, dormant structures, creating a persistent source of infection (Noble and Coventry, 2005; Pérez-Jaramillo et al., 2016). While chemical pesticides are common, they are environmentally damaging, and lead to resistant pathogens, creating a need for sustainable alternatives (Lamichhane et al., 2018; Strange and Scott, 2005). This problem is worsened by soil health decline. Intensive farming practices reduce soil microbial diversity and fertility, lowering yields and ecosystem resilience (Geisseler and Scow, 2014; Wu et al., 2011; Xuan et al., 2012). Although integrated pest management (IPM) strategies are used, their success is limited by an incomplete understanding of the complex soil environment where these diseases occur (Abawi and Widmer, 2000; Bailey and Lazarovits, 2003; Van Bruggen and Finckh, 2016).

The soil microbiome, particularly in the rhizosphere, is vital for plant health, managing both helpful and harmful microbes (Dubey et al., 2019; Wieland et al., 2001). Therefore, there is a rising awareness that improving soil health and understanding microbial community changes are necessary for developing new agricultural practices that improve productivity and environmental care (Blaya et al., 2015; Mendes et al., 2011; Jansson and Hofmockel, 2020). The rise of high throughput next generation sequencing (NGS) has revolutionized our ability to probe the immense complexity of the soil ecosystem. Metagenomics, encompassing both amplicon and shotgun sequencing, has provided an unprecedented window into the structural composition and functional potential of soil and rhizosphere microbiomes, moving beyond culturable isolates to reveal the vast, untapped reservoir of microbial diversity (Gore-Lloyd et al., 2019; Mendes et al., 2014). This approach has been successfully employed to characterize the microbiomes associated with disease suppressive soils, identifying specific microbial consortia linked to the suppression of pathogens (Carrión et al., 2019; Wang et al., 2024), and great amplified when integrated with other meta omics technologies. This approach is further amplified by the *de novo* assembly of complete genomes from isolated beneficial strains, which provides a genetic blueprint for elucidating direct causal effects, such as the specific genes or pathways involved in pathogen antagonism like chitinases, lipopeptides, and other antimicrobial compounds (Levy et al., 2018; Hesse et al., 2018).

The microbiome diversity involved in the suppression of some plant pathogens like *Rhizoctonia solani* AG8 (Yin et al., 2013; Penton et al., 2014) has been figured out using DNA based community profiling techniques such as sequencing or microarrays (Kyselková et al., 2009; Mendes et al., 2011; Klein et al., 2013; Cha et al., 2016). These approaches have been used to characterize the community genetic potential and metatranscriptomics to identify the differential gene expression and active metabolic processes of microbial communities in suppressive soils remains novel and presents a significant opportunity to identify the complex microbial mechanisms directly from complex soil samples (Wieland et al., 2001; Blaya et al., 2015; Mendes et al., 2014; Wang et al., 2024), thereby identifying the actual players carrying out suppressive functions *in situ* (Chapelle et al., 2016; Zhang et al., 2019), hence creating a powerful pipeline for linking microbial community structure to function, allowing a detailed understanding of their interactions (Agler et al., 2016; Toju et al., 2018). However, these advanced approaches are predicated on a critical, and often missing, foundational element. Without this ecological context, the identification of truly relevant suppressive taxa and the design of biologically meaningful functional assays remain profoundly challenging. This major lack of knowledge is clearly shown in tobacco black shank, caused by *Phytophthora nicotianae*. Since 1896, it has become a main limit on tobacco farming in over 120 countries, causing large economic losses (Toju et al., 2018; Panabieres et al., 2016; Kamoun et al., 2015). The pathogen attacks many plants and has a damaging life cycle, invading all plant parts and causing tissue death, yellowing, poor growth, and wilting (Csinos, 2005; Lamour, 2013). Its management is difficult due to its ability to survive saprophytically in soil for 4–6 years *via* long-lived oospores, and its capacity for explosive population growth facilitated by a rapid 72-hour reproductive cycle (Erwin and Ribeiro, 1996; Shew, 1983; Bowers and Mitchell, 1991). These traits often render traditional strategies like crop rotation and chemical control inadequate, strengthening the necessity for novel, ecology-based management solutions.

Therefore, to bridge the gap between ecological surveys and mechanistic studies, we implemented a six-year soil sampling regime in monoculture tobacco fields infected with *P. nicotianae*. We hypothesized that temporal changes in *P. nicotianae* population genetics and abundance, coupled with concurrent assembly of the soil microbiome, are critical determinants of disease. Using long-term metagenomics, we analyzed pathogen population dynamics, revealing significant seasonal shifts in abundance and structure. Simultaneously, we identified key microbial successional patterns and specific keystone taxa whose functional gene profiles correlated with disease suppression. These findings provide an ecological and genomic framework that elucidates the drivers of disease and establishes a foundational dataset for future biocontrol agent isolation, genome mining, and validation in gnotobiotic systems. This time resolved perspective is essential for developing predictive, microbiome informed strategies to manage tobacco black shank.

## 2 Materials and methods

### 2.1 Study site, design, and agronomic management

A six-year longitudinal study was conducted in commercial tobacco fields in Dali, Yunnan, China, to investigate temporal dynamics of the soil pathobiome. The region has a temperate climate with distinct seasons, cold winters, hot, dry summers, and an average annual rainfall of 840 mm. The selected fields had a documented history of black shank and were maintained under monoculture with tobacco cv. K326 for six consecutive years during the sampling period. Soil samples were collected annually to monitor pathogen inoculum. All agronomic practices, including fertilizer applications, adhered to the national standard management protocol (China, GB 21117-2017).

### 2.2 Soil sampling and pathogen monitoring

Longitudinal soil sampling was conducted over six consecutive years to capture temporal dynamics of both the soil microbiome and the target pathogen, *P. nicotianae*. Sampling was done at the tobacco vegetative growth stage (60 days), following initial black shank symptom appearance. A stratified random sampling design was employed to ensure representation of field heterogeneity, using five replicates each of 100 m². From each plot, three composite rhizosphere samples were collected from 10 randomly selected plants (15–20 cm depth), intentionally spanning the interior and periphery of disease patches. Soil was shaken from roots, homogenized, transported on ice, and air dried for analysis. Replicate samples were submitted to Wuhan Seqhealth Technology Co., Ltd, China (https://www.seqhealth.cn/lxwm) for DNA extraction and metagenomic sequencing. Concurrently, separate sampling quantified the *P. nicotianae* inoculum pool at three critical stages: pre-sowing, vegetative growth, and harvest. Both rhizospheric and bulk soil were collected. Pathogen isolation employed selective media and baiting techniques (Ferguson and Jeffers, 1999; Erwin et al., 1983), with morphological identification based on established taxonomic keys (Waterhouse, 1970; Louca et al., 2018).

### 2.3 Metagenomic analysis of soil microbiome

DNA libraries from six annual samples (YC_1, XM_1, MHT_1, LD_1, SD_1 and YJ_1) were prepared and sequenced on MGISEQ-T7, generating 150 bp paired-end (PE) reads. Raw reads were quality-checked with FastQC (v0.11.9) (Andrews, 2010), adapter and low quality bases were removed using Trimmomatic (v0.39) (Bolger et al., 2014) with parameters: ILLUMINACLIP:adapters.fa:2:30:10, LEADING:3, TRAILING:3, SLIDINGWINDOW:4:15, and MINLEN:36. This filtered reads with ≥10% undetermined bases or over 50% of bases with Q ≤ 10. Post-processing, all samples had high quality data (Q30 > 95.3%; >99.9% read retention). The assembly of the high quality clean reads from each sample were assembled *de novo* using MEGAHIT (v1.1.2) (Li et al., 2015a), with multiple *k-mers* (57 to 137). The optimal assembly was selected based on contig statistics, and open reading frames (ORFs) were predicted from the assembled contigs using Prokka (v1.13.3) (Seemann, 2014). Predicted genes shorter than 400 bp were filtered out, and the nucleotide sequences were translated. Amino acid sequences from all samples were clustered using CD-HIT (v4.7) (Li and Godzik, 2006) with identity and coverage thresholds of 95% and 90%, respectively, resulting in a final catalogue of 1,835,559 non-redundant genes. For gene abundance profiling clean reads from each sample were mapped to non-redundant gene catalogue using Bowtie2 (v2.3.3.1) (Langmead and Salzberg, 2012). The gene abundance in each sample was calculated using the following formula to yield Reads Per Kilobase per Million mapped reads (RPKM):


$$\text{RPKM}=\frac{\text{Number of reads mapped to gene}}{\text{Gene length }(\text{kb})\times \text{Total million mapped reads}}\times 10^{9}$$

This generated a gene abundance matrix across all samples, which was used for subsequent analyses.

### 2.4 Differential gene expression analysis

Differentially abundant genes were identified using Wilcoxon rank-sum tests for comparing transcript levels across years with significance thresholds set at an absolute *log_2_* fold change (*log_2_*FC) > 1.0 and *p-*value *< 0.05*. Genes were categorized into highly significant (*p < 0.001* and *log_2_*FC > 2), very significant (*p < 0.01* and *log_2_*FC > 1.5), significant up (*p < 0.05* and *log_2_*FC > 1.0), significant down (p *< 0.05* and *log_2_*FC < -1.0), marginally significant (*p < 0.1*) and not significant (*p ≥ 0.1*). The results were visualized using volcano plots generated using *ggplot2* (version 4.0.0) (Wickham, 2016) in R v.4.5.2 (R Core Team, 2000), a pseudocount of 0.0001 was added to ensure numerical stability in fold change calculations.

### 2.5 KEGG pathway and functional enrichment analysis

Functional profiling of metagenomic data was conducted through KEGG orthology (KO) analysis to elucidate temporal shifts in metabolic potential. A total of 3,629 KOs, representing 387 metabolic pathways, were analyzed across the six-year sampling period. The analysis employed a multi-faceted approach to characterize pathway dynamics: (1) assessing temporal progression via density distribution of *log_2_* fold changes; (2) visualizing expression patterns of variable KOs through hierarchical clustering of Z-score normalized abundances; and (3) conducting comparative pathway enrichment analysis.

### 2.6 Antibiotic resistance gene profiling

The soil antibiotic resistance gene (ARG) profiling and resistome dynamics was characterized through annotation against the Antibiotic Resistance Genes Database (ARDB) (https://ardb.cbcb.umd.edu/). We profiled 1,692-1,759 genes across six year-over-year comparisons, categorizing 73 distinct mechanisms into 12 major classes. Differential abundance analysis between consecutive growing seasons was performed using DESeq2 (Love et al., 2014). Significant changes were defined as | *log_2_*FC| > 1 with an adjusted *p-*value *< 0.05* (Benjamini and Hochberg, 1995). Community-wide coordination of resistance genes was evaluated using linear regression, with slope and R² values indicating response synchronization.

### 2.7 Virulence factor profiling and categorization

Virulence factors were profiled from tobacco soil metagenomes across six annual comparisons. Initial screening identified 67,227 virulence associated genes to 14,317 *Phytophthora* relevant virulence factors (21.3% retention) using species specific criteria to focus on oomycete pathogens relevant to tobacco soil ecosystems. Virulence factors were systematically classified into ten functional categories based on their annotated mechanisms: (1) Motility and Adhesion (flagella, pili, fimbriae), (2) Secretion Systems (T3SS, T4SS, T6SS), (3) Transport and Metabolism (ABC transporters, nutrient acquisition), (4) Regulatory Systems (two-component systems, signaling), (5) Stress Response (heat shock, oxidative stress), (6) Effectors and Toxins, (7) Structural Components (LPS, capsules), (8) Nutrient Acquisition, (9) Host Interaction, and (10) Other Virulence Factors. Persistent virulence factors (present in ≥4 of 6 comparisons) were identified and evaluated using a persistence score incorporating fold change magnitude, directional consistency, and occurrence frequency.

### 2.8 Carbohydrate-active enZymes profiling

Our analysis encompassed 27,886 significant carbohydrate-active enZymes (CAZy) profiling and network analysis genes identified across five comparative transcriptomic datasets. Genes were annotated according to the CAZy database (https://www.cazy.org/) into six enZyme classes. A multi-tiered analytical approach identified variable enZyme families through a composite score incorporating coefficient of variation, abundance range, and max/min ratios. Co-occurrence networks were constructed using correlation thresholds (r > 0.6), with patterns classified by expression trajectories across the study period.

### 2.9 COG functional annotation

Metagenomic sequencing data were annotated against the COG (Clusters of Orthologous Groups) database (https://www.ncbi.nlm.nih.gov/research/cog), encompassed 479,857 unique genes distributed across 132 distinct COG functional categories. Relationships between COG categories were examined through correlation-based distance matrices and hierarchical clustering (Ward.D2 method). Linear regression models quantified changes in gene counts and abundances across sampling points. Combined correlation matrices identified co-varying functional modules and potential networks within the microbial community.

### 2.10 Taxonomic profiling and diversity

Taxonomic annotation was performed by aligning predicted protein sequences against the NCBI non-redundant database using DIAMOND v2.0.11 (Buchfink et al., 2021) (E-value < 1e-5). The generated abundance profiles across seven taxonomic levels, identifying 66 phyla, 555 genera, and 717 species. Samples were grouped into three periods (Years 1-2, 3-4, 5-6) for analysis. Alpha diversity metrics (richness, Shannon, Simpson indices) were calculated using the *vegan* package after normalizing to proportional abundances. Temporal trends were assessed through linear regression with Shannon diversity as the response variable, while group comparisons employed ANOVA. Beta diversity was quantified using Bray-Curtis dissimilarity distance with PCoA and hierarchical clustering. Distance-decay relationships were evaluated using Mantel tests. All visualizations, including faceted bar plots and heatmaps of top taxa, were arranged using the *patchwork* package.

## 3 Results

### 3.1 Comparative gene expression analysis across consecutive years

**Figure 1:**
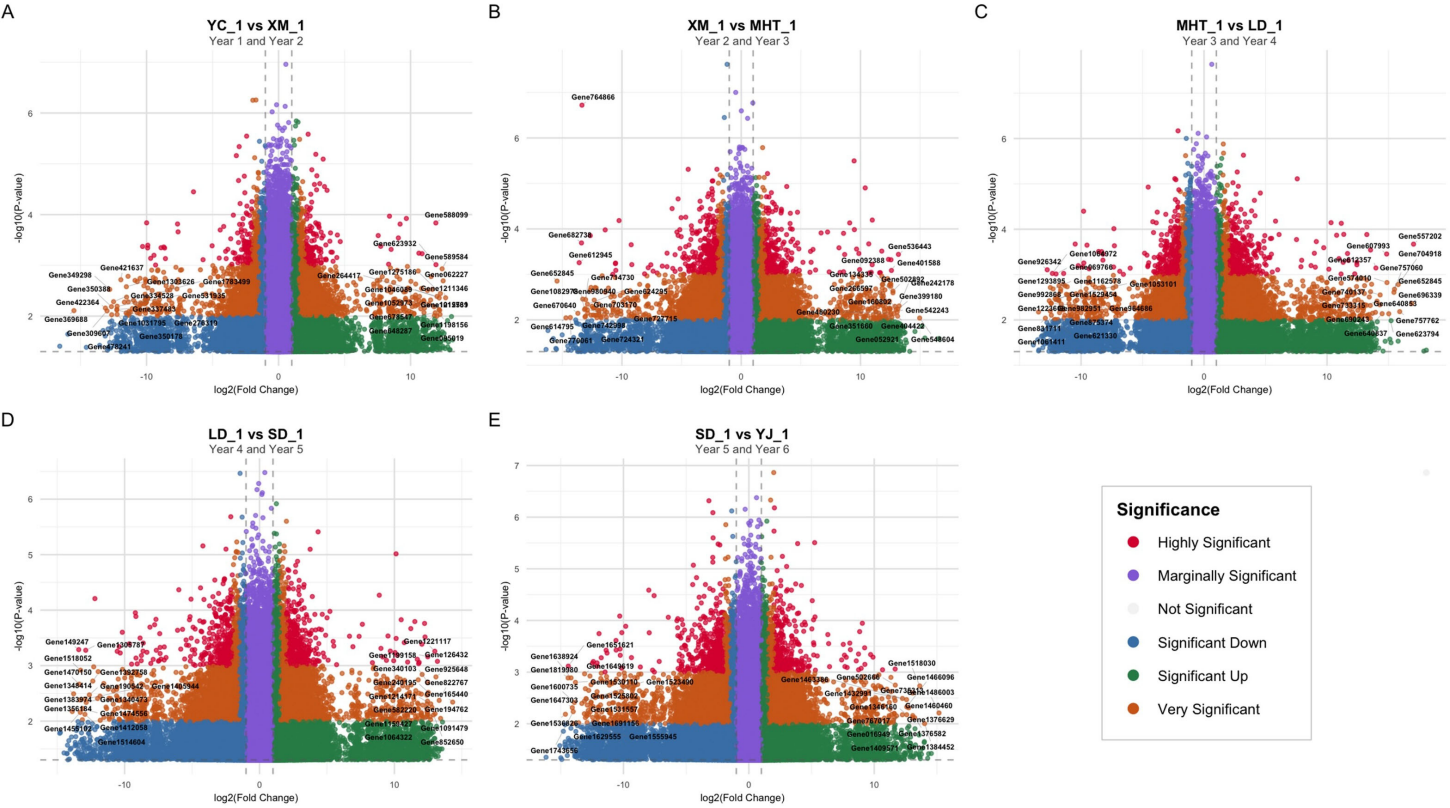
Comparative volcano plots of differential gene expression across five years+: **(A)** Year YC_1 vs XM_1 (Year 1 vs 2), **(B)** Year XM_1 vs MHT_1 (Year 2 vs 3), **(C)** Year MHT_1 vs LD_1 (Year 3 vs 4), **(D)** Year LD_1 vs SD_1 (Year 4 vs 5), and **(E)** Year SD_1 vs YJ_1 (Year 5 vs 6). Each point represents a gene, colored by significance category. Dashed lines indicate significance thresholds (|*log*_2_FC|=1, *p* = 0.05).

Longitudinal transcriptomic profiling revealed substantial annual variation with ~255,000 genes examined per comparison. The initial annual transition YC_1 vs XM_1 comparison (Year 1 vs 2, **Figure 1A**) identified 29,484 upregulated and 26,339 downregulated genes. The XM_1 vs MHT_1 transition (Year 2 vs 3, **Figure 1B**) showed 42,134 upregulated and 43,010 downregulated genes. MHT_1 versus LD_1 (Year 3 vs 4, **Figure 1C**) revealed 44,682 upregulated and 44,001 downregulated genes. The LD_1 vs SD_1 comparison (Year 4 vs 5, **Figure 1D**) exhibited the highest number of upregulated genes (60,073) with 43,828 downregulated genes. SD_1 versus YJ_1 (Year 5 vs 6, **Figure 1E**) demonstrated balanced regulation with 46,806 upregulated and 47,310 downregulated genes. The cumulative six-year comparison revealed 58,203 upregulated and 43,083 downregulated genes. Highly significant genes (*p < 0.001*, | *log_2_*FC| > 2) progressively increased from 268 in Year 1–2 to 1,006 in Year 1-6, indicating substantial transcriptional reprogramming.

### 3.2 KEGG pathway analysis reveals metabolic shifts

**Figure 2:**
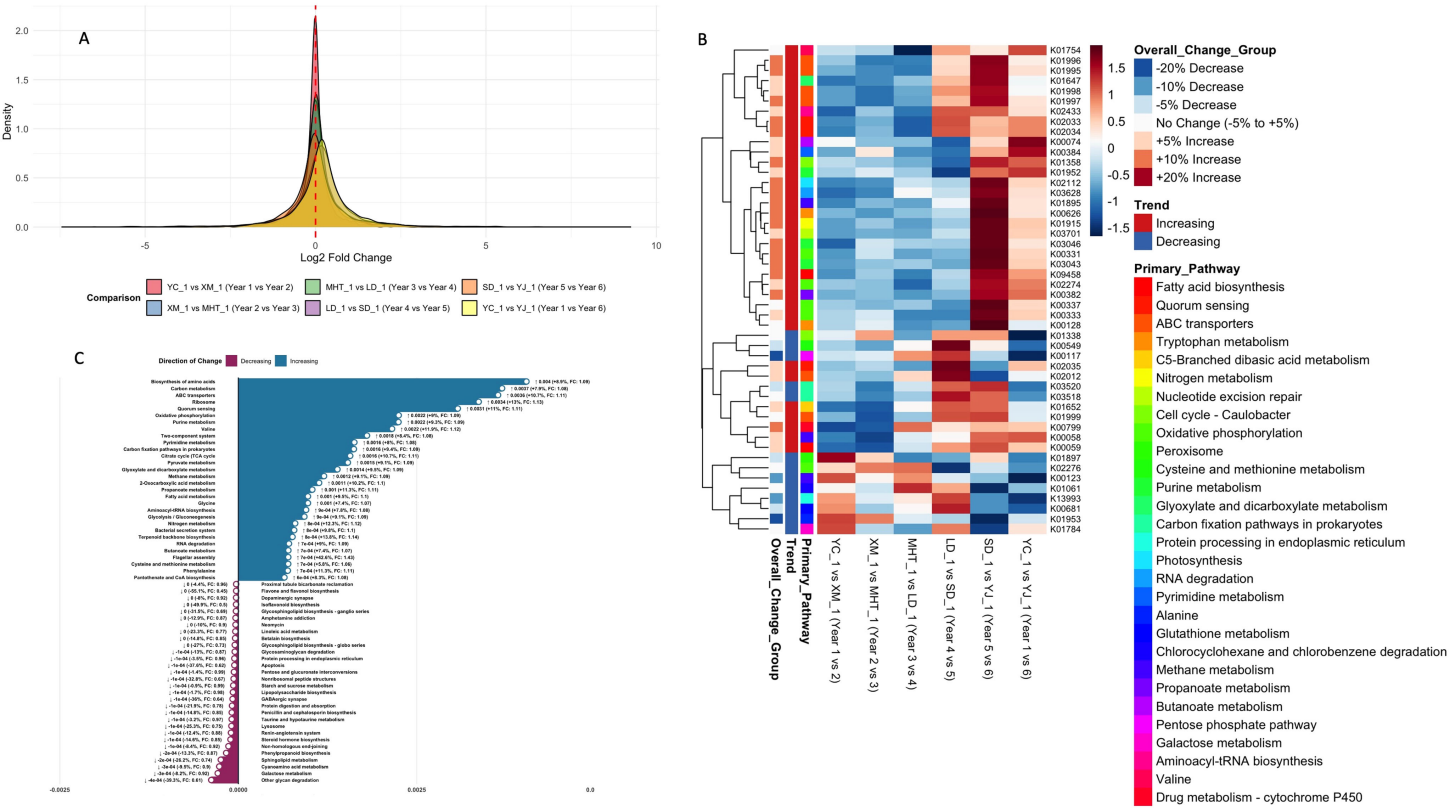
Progressive shifts in density distributions, KO abundance, and pathway divergence over a six-year period. **(A)** Density distributions comparing all six-year comparisons simultaneously, showing progressive shifts in distribution patterns across the sequential gradient. **(B)** Heatmap of Z-score normalized for top KOs abundance across year comparisons. Rows represent individual KOs, columns represent sequential year comparisons from Year 1–2 to Year 1-6. Annotation bars indicate primary pathway affiliation (29 pathways represented), overall trend (increasing/decreasing between Year 1-6), and percentage change categories. **(C)** Top 60 altered KEGG pathways between early (Years 1-3) and late (Years 4-6) sampling periods. Bar plot displaying the most increased and decreased pathways based on abundance changes, sorted by magnitude of change. Positive values indicate higher abundance in late years.

Analysis of 3,629 KOs revealed substantial functional reorganization, with distinct patterns emerging across sequential annual comparisons (**Supplementary Table S1**). The percentage of increasing KOs rose from 46.8% in Year 1–2 to 66.7% in the cumulative six Year comparison. The Year 4–5 transition showed the highest proportion of increasing KOs (64.8%) among consecutive comparisons, while Year 5–6 exhibited more balanced distribution (46.7% increasing) (**Figure 2A**). Average *log_2_*FC progressed from negative (-0.023) in early years to positive (+0.247) later, indicating an inflection point in microbial metabolism (**Supplementary Figure S1**). Heatmap analysis of the top KOs across 29 primary pathways revealed coordinated functional shifts (**Figure 2B**) with ABC transporters constituted the largest functional group (6 KOs), followed by oxidative phosphorylation (5 KOs) and methane metabolism (3 KOs). Individual KOs governing quorum sensing (K02034) and fatty acid biosynthesis (K00059) increased by 15.2% and 9.7%, respectively, indicating enhanced microbial coordination and lipid metabolism over time. Comparative enrichment analysis between early (Years 1-3) and late (Years 4-6) periods quantified this metabolic reprogramming, identifying 317 pathways with significantly increased activity versus only 70 with decreased activity (**Figure 2C**) (**Supplementary Table S1**). The most substantially increased pathways in late years included biosynthesis of amino acids (+8.9%, FC: 1.09), carbon metabolism (+7.9%, FC: 1.08), ABC transporters (+10.7%, FC: 1.11), ribosome (+13.0%, FC: 1.13), and quorum sensing (+11.0%, FC: 1.11). The most decreased pathways in late years included other glycan degradation (-39.3%, FC: 0.61), galactose metabolism (-8.2%, FC: 0.92), cyanoamino acid metabolism (-9.5%, FC: 0.90), and sphingolipid metabolism (-26.2%, FC: 0.74). This systematic shift toward anabolic capacity and cellular signaling, coupled with reduced complex carbohydrate metabolism, suggests a functional maturation of the soil microbiome toward a more biosynthetically active state.

### 3.3 Antibiotic resistance dynamics in *Phytophthora* infected tobacco soils

**Figure 3:**
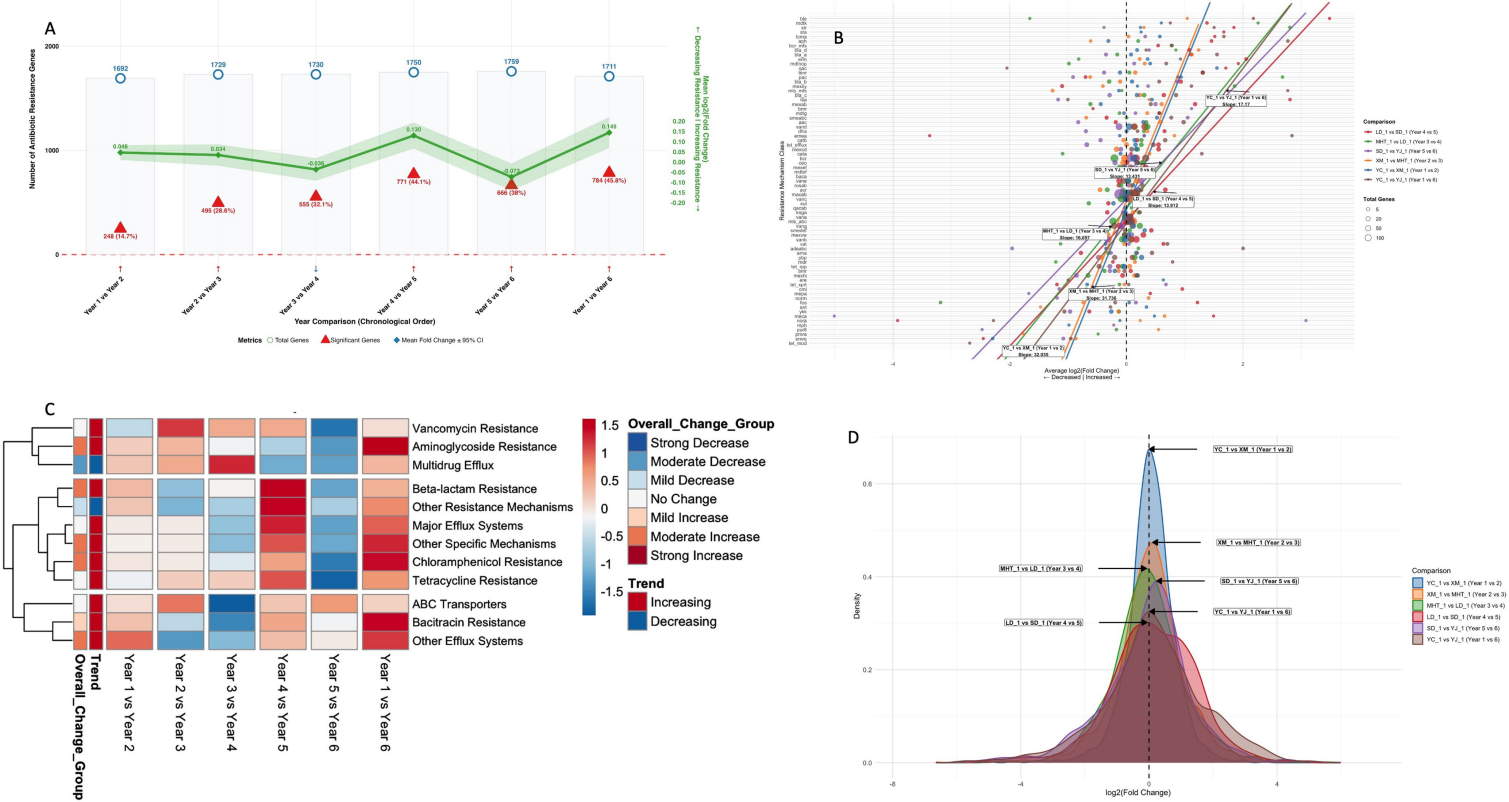
Antibiotic resistance gene dynamics in *phytophthora*-infected tobacco soils. **(A)** Density distributions of *log_2_*FC for all resistance genes across consecutive all year’s comparison and the cumulative six-year period. **(B)** Coordination of resistance mechanisms, showing linear relationships (slope and R²) between gene abundance and fold change for each comparison. **(C)** Z-scores of major antibiotic resistance classes across year comparisons, indicating relative upregulation (red) or downregulation (blue). **(D)** Density plots of significant resistance gene fold changes, illustrating shifts in the response distribution of the microbial community over time.

Resistome analysis of 1,692-1,759 antibiotic resistance genes revealed dynamic microbial adaptations under persistent *Phytophthora* pressure (**Supplementary Table S2**). The initial transition (Year 1-2) established a baseline resistance profile with moderate increases (mean +0.046 *log_2_*FC) and limited significant changes (14.7% of genes), as visualized in the comparative density distributions (**Figure 3A**). In Year 2-3, where microbial communities demonstrated their most synchronized defense response (slope = 31.736, R² = 0.825, **Figure 3B**). This period featured strategic upregulation of beta-lactamase systems (bla_d: +1.239 *log_2_*FC, bla_a: +1.125) and multidrug efflux pumps (mexab: +0.604), representing organized microbial preparation against escalating *Phytophthora* challenge. The subsequent Year 3–4 transition marked a dramatic resistance collapse, showing the most substantial antibiotic resistance gene suppression among all consecutive comparisons with the highest number of downregulated genes (n=917) and negative meanFC (-0.037, **Figure 3A**). This vulnerability window exhibited severe coordination breakdown (R² = 0.674) and comprehensive defense failure across multiple systems. The heatmap analysis reveals widespread suppression (**Figure 3C**), particularly affecting multidrug resistance systems (mdr: -1.211 *log_2_*FC), fosfomycin resistance (fos: -3.187 *log_2_*FC), and ABC transporters (-1.935 Z-score), effectively compromising microbial defense against *Phytophthora*-produced antimicrobial compounds. A targeted recovery phase followed in Year 4-5, demonstrating microbial resilience through the second-highest resistance increase (mean +0.130 *log_2_*FC) and restored coordination (R² = 0.796). The recovery showed mechanism-specific patterns, with extreme increases in bleomycin resistance (ble: +3.484 *log_2_*FC) suggesting oxidative stress adaptation, while β-lactam resistance showed the strongest Z-score recovery (+1.525, **Figure 3C**) and major efflux systems rebounded (+1.310 Z-score), restoring antimicrobial compound export capacity. The final year transition (Year 5-6) indicated ongoing instability, returning to negative mean fold change (-0.073 *log_2_*FC) despite high gene significance (38.0%, **Supplementary Table S3**), suggesting continued microbial community fluctuations in response to persistent *Phytophthora* pressure (**Figure 3D**). The cumulative six-year perspective (Year 1-6) revealed the adaptation patterns, with the highest overall resistance increase (mean +0.145 *log_2_*FC) and substantial gene alterations (45.8% significant) (**Figure 3A**). The density distribution shows progressive right shifting of resistance genes (**Figure 3A**, 3D), while long-term adaptation featured sustained upregulation of aminoglycoside resistance (aph: +2.769 *log_2_*FC), MLS resistance (emea: +2.840 *log_2_*FC), and strategic multidrug systems (adeabc: +2.001 *log_2_*FC). Despite achieving high coordination (R² = 0.838) (**Figure 3B**), the six-year trajectory demonstrates continued microbial community restructuring rather than stabilization. This sustained coordination occurred alongside a net increase in antibiotic resistance (mean +0.145 *log_2_*FC), indicating that the soil microbiome remains in a state of dynamic, coordinated adaptation directly driven by the persistent selective pressure of the *Phytophthora* pathogen.

### 3.4 Virulence factor evolution under monoculture pressure

**Figure 4:**
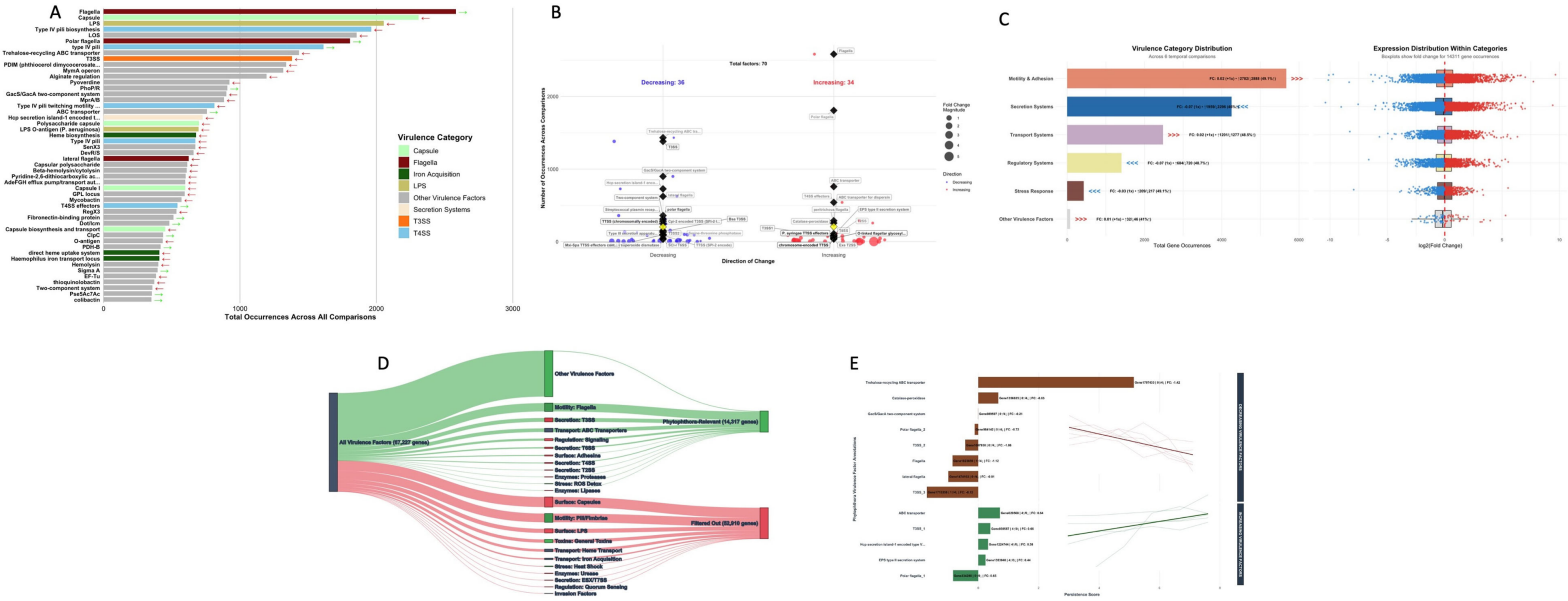
Temporal dynamics of virulence factors across six-year tobacco monoculture system. **(A)** Abundance of major virulence factors, showing category distribution and expression trends. **(B)** Directional changes in virulence factors, displaying variation magnitude and frequently observed elements. **(C)** Comparative analysis of virulence factor categories, showing both abundance distribution and expression patterns. **(D)** Flow visualization of virulence factor filtering from comprehensive database to *Phytophthora*-specific analysis. **(E)** Consistently present virulence factors across multiple sampling points, ranked by persistence score and showing expression direction.

Analysis of 14,317 virulence factor occurrences revealed flagella as most abundant (2,583 occurrences), followed by capsule synthesis (2,309) and LPS components (2,054) (**Figure 4A**, **Supplementary Table S3**). Type IV pili systems were well represented (1,962 occurrences), highlighting adhesion importance. While flagella increased (average *log_2_*FC = +0.063), LPS and capsule systems declined slightly. Among the top 20 virulence factors, only five showed increasing trends, while fifteen exhibited decreasing patterns. The distribution of virulence factor directional changes revealed a nearly equal split between increasing (48.6%, n=34) and decreasing (51.4%, n=36) trends across the 70 unique virulence factors identified (**Figure 4B**). FC magnitude analysis indicated moderate regulatory changes overall (average |FC| = 0.39), with increasing factors demonstrating higher magnitude changes (average |FC| = 0.547) compared to decreasing factors (average |FC| = 0.243). The maximum observed fold change magnitude reached 5.307, indicating substantial regulation of specific virulence mechanisms, while the minimum was 0.002, representing near constitutive expression patterns. High count virulence factors (>30 occurrences) revealed important functional patterns: ABC transporters (758 occurrences, FC = +0.047) and ABC transporters for dispersin (288 occurrences, FC = +0.063) showed consistent increasing trends, while trehalose-recycling ABC transporters (1,432 occurrences, FC = -0.004) and T3SS systems (1,381 occurrences, FC = -0.162) demonstrated decreasing patterns. Flagella related systems showed variable regulation, with general flagella increasing (FC = +0.063) but polar flagella subtypes showing both increasing (FC = +0.003) and decreasing (FC = -0.178) trends. Functional categorization of the 14,311 gene occurrences across six major categories revealed motility and adhesion as the dominant functional group (5,670 occurrences, 39.6% of total), exhibiting nearly balanced regulation (49.1% upregulated, 50.9% downregulated) with a slight increasing trend (average FC = +0.022) (**Figure 4C**). Secretion Systems represented the second largest category (4,255 occurrences, 29.7% of total), displayed an overall decreasing pattern (46% upregulated, average FC = -0.070), thereby suggesting potential evolutionary constraints on these energy intensive mechanisms. Transport (2,478 occurrences, 17.3% of total) maintained balanced regulation (48.5% upregulated, average FC = +0.020), while Regulatory Systems (1,404 occurrences, 9.8% of total) demonstrated decreasing trends (48.7% upregulated, average FC = -0.067). Stress Response mechanisms (426 occurrences, 3.0% of total) preserved near equilibrium regulation (49.1% upregulated, average FC = -0.034). The filtering workflow from comprehensive virulence factors to *Phytophthora* specific analysis retained 14,317 genes (21.3% retention), excluding 52,910 genes (78.7%) irrelevant to oomycete pathogens (**Figure 4D**). This targeted approach ensured biological relevance to tobacco soil ecosystems where *Phytophthora* species represent significant pathogenic threats. The retained factors predominantly involved adhesion mechanisms, secretion systems, and metabolic adaptations characteristic of oomycete pathogenesis. Persistence analysis of 10,127 virulence factors identified 87 factors (0.9%) that demonstrated consistent presence across ≥4 temporal comparisons, revealing distinct directional patterns among these temporally stable elements (**Figure 4E**). The trehalose recycling ABC transporter demonstrated the highest persistence score (5.148) with strong decreasing consistency (FC = -1.422, 0↑/4↓), while flagella systems showed substantial persistence (score = 1.007) with predominantly decreasing trends (FC = -1.063, 1↑/3↓). Conversely, ABC transporters exhibited strong increasing persistence (score = 0.716, FC = +0.635, 4↑/0↓), followed by T3SS systems (score = 0.400, FC = +0.656, 4↑/0↓) and Type VI Secretion Systems (H-T6SS, score = 0.322, FC = +0.579, 4↑/0↓). Catalase-peroxidase systems showed strong decreasing persistence (score = 0.663, FC = -0.631, 0↑/4↓), while GacS/GacA two-component regulatory systems displayed complex gene variant specific persistence (scores ranging from 0.177 to -0.004), highlighting the complexity of regulatory network modifications for adaptive responses to long-term biotic pressures.

### 3.5 Network analysis of CAZy families

**Figure 5:**
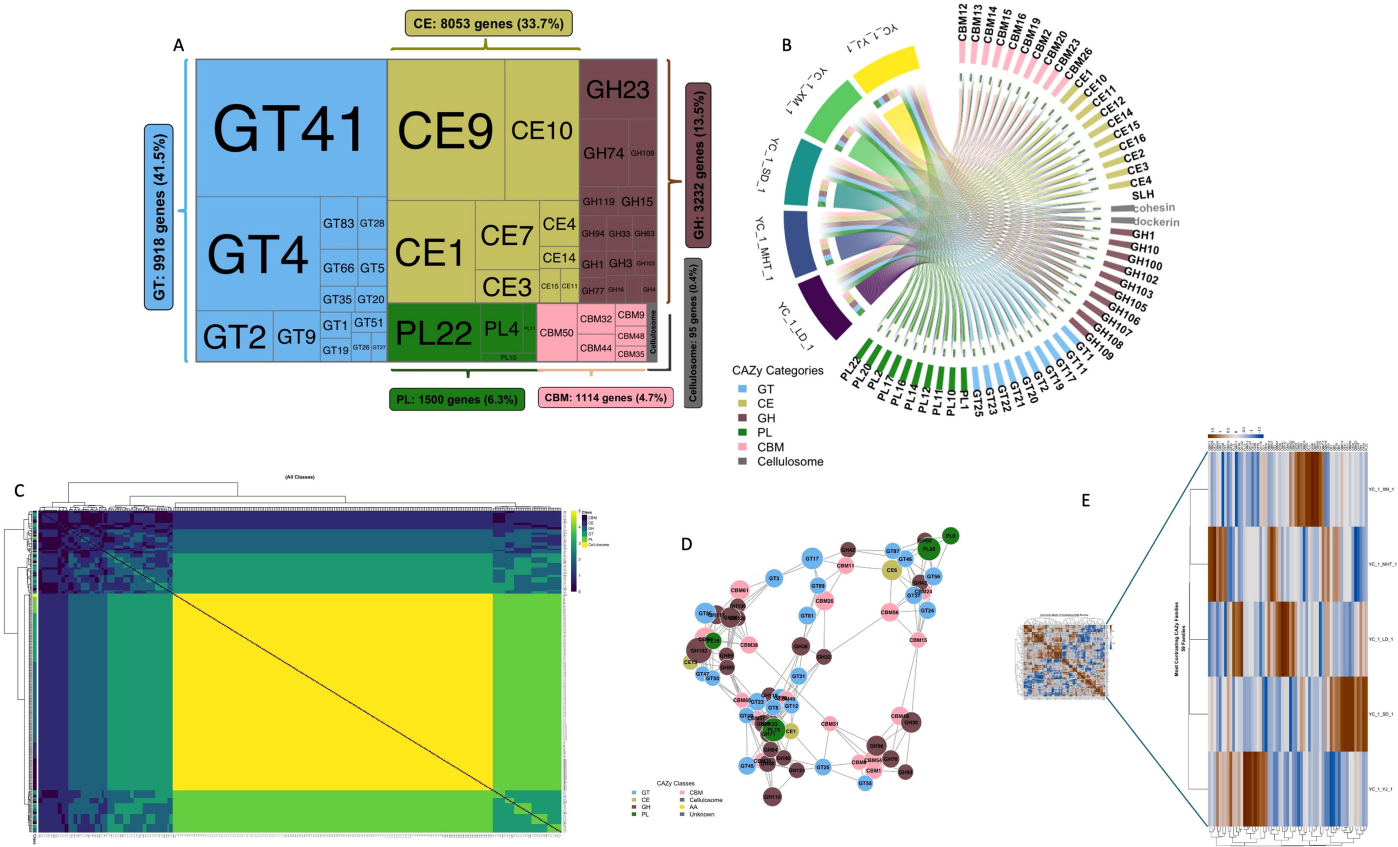
CAZy family distribution and network architecture across six years. **(A)** Distribution of dominant CAZy families, showing abundance patterns across enZyme classes. **(B)** Persistence patterns of top CAZy families across annual comparisons, showing universal conservation of CBM families. **(C)** Co-occurrence patterns among contrasting CAZy families, revealing coordinated expression modules. **(D)** Network topology of 70 contrasting families, with node size representing contrast score, color indicating enZyme class, and edge thickness showing co-occurrence strength. **(E)** Heatmap analysis of co-occurrence patterns among 59 contrasting families.

Analysis of 27,886 significant CAZy genes revealed remarkably conserved class-level architecture, with CV ranging from just 1.2% for Carbohydrate Esterases (CE) to 5.8% for Cellulosome components (**Supplementary Table S4**). Glycosyl Transferases (GT) maintained consistent dominance (38.8 ± 0.5%), followed by CE (29.6 ± 0.4%), Glycoside Hydrolases (GH) (18.3 ± 0.4%), Carbohydrate-Binding Modules (CBM) (6.4 ± 0.2%), and Polysaccharide Lyases (PL) (6.1 ± 0.2%). This stability at the class level masked substantial family-level reorganization, with 59 contrasting families (19.9% of total) exhibiting significant variability. The top 50 families represented 23,912 genes (85.7% of total), with extreme dominance observed in the top 10 families (GT41: 4,505 genes; CE9: 2,850 genes; GT4: 2,427 genes) collectively representing 70.6% of this subset (**Figure 5A**). Chord diagram analysis revealed universal persistence of all CBM families (CBM12, CBM13, CBM14, CBM15, CBM16, CBM19, CBM2, CBM20, CBM23, CBM26) across all six years, indicating their fundamental role in carbohydrate recognition (**Figure 5B**). In contrast, 59 families demonstrated high variability, with contrast scores ranging from 0.230-0.497, with CBM emerged as the most variable class (21 contrasting families, 35.6%), followed by GT (25.4%), GH (22.0%), CE (10.2%), and PL (6.8%) (**Supplementary Table S4**). The most contrasting families included GH102 (0.497), CBM4 (0.437), and PL15 (0.437), specializing in peptidoglycan lytic transglycosylase activity, cellulose/xyloglucan binding, and oligo-alginate lyase function, respectively. Heatmap analysis revealed that while overall co-occurrence patterns remained stable (**Figure 5C**), contrasting families showed distinct expression trajectories, with GH families exhibiting the highest mean contrast scores (0.305), followed by PL (0.273) and GT (0.270) (**Supplementary Figures S2A–E**).

Network analysis of 70 contrasting families revealed substantial interconnectivity (404 edges, network density 0.167, average node degree 11.5), indicating coordinated modular operation rather than independent variation (**Figure 5D**). GH families formed the largest network component (25 nodes), followed by GT (21 nodes) and CBM (17 nodes). GH102, CBM4, and PL15 emerged as key network hubs, suggesting central roles in coordinating temporal adaptations. Pattern analysis classified 59 families into five distinct adaptation clusters: High-Variability Specialists (16 families, CV: 0.770), Moderate Adaptors (23 families), Stable High-Contrast (10 families), High-Abundance Stable (6 families), and Sporadic Variables (4 families). The strong correlation between Contrast_Score and CV_Abundance (r = 0.907) confirmed coordinated expression modulation. Annual progression revealed clear temporal specialization, with early years (Year 1-2) showing mixed variability clusters and the mature phase (Year 1-6) dominated by extreme contrast specialists, demonstrating progressive functional optimization under persistent environmental pressure (**Figure 5E**). Heatmap analysis showed stable overall co-occurrence patterns (**Figure 5C**) with distinct class specific networks (**Supplementary Figure S2**).

### 3.6 Genetic functional decoupling in COG profiles

**Figure 6:**
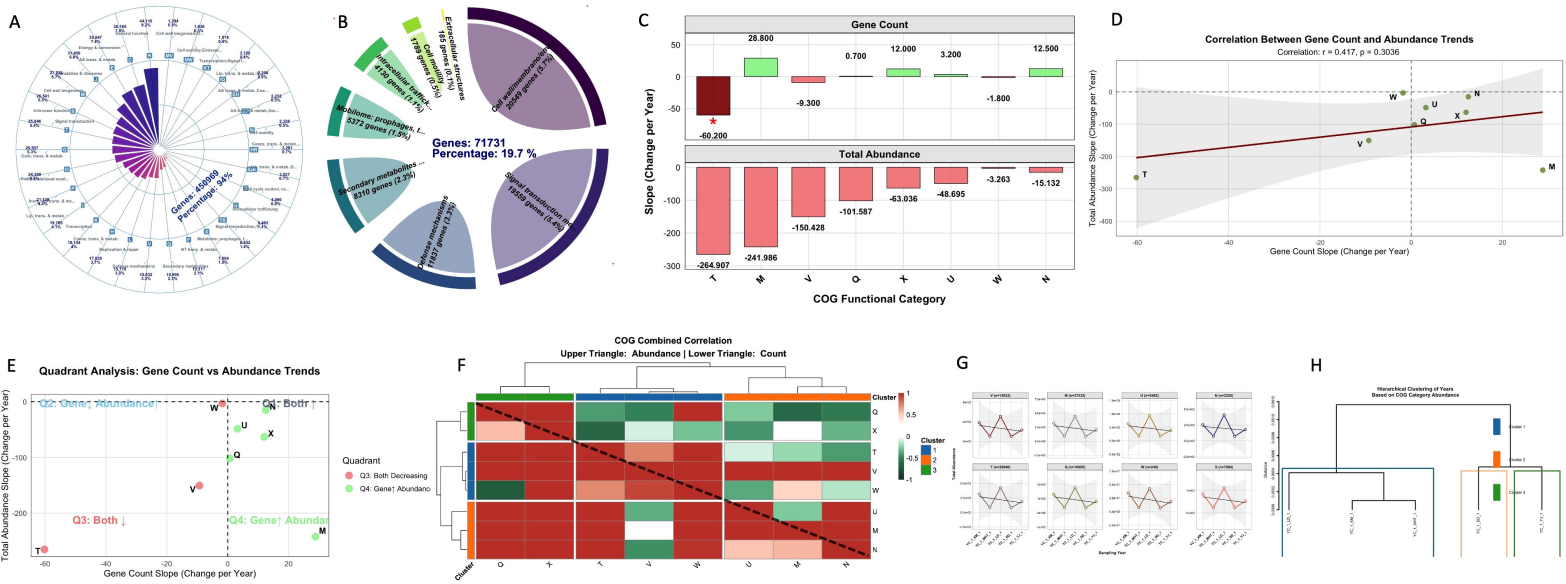
Functional metagenomic analysis of COG. **(A)** Distribution of major COG functional categories showing 450,969 gene occurrences (94% coverage). **(B)** Chord diagram of eight core COG category relationships. **(C)** Abundance trends of eight core COG categories with linear regression fits. **(D)** Correlation between gene count and abundance trends (r = 0.417). **(E)** Quadrant analysis showing divergent “Gene↑ Abundance↓” patterns and consonant decreases. **(F)** Comparative analysis of gene count versus abundance trends. **(G)** Temporal trajectory of total functional abundance with YC_1_LD_1 as critical transition phase. **(H)** Hierarchical clustering of temporal samples based on COG abundance profiles.

Analysis of 479,875 genes across 132 categories revealed distinct functional profiles characterized by both single and multi-functional COG categories. The distribution encompassed 30 major COGs representing 450,969 gene occurrences (94% coverage), with all categories demonstrating high persistence across ≥80% of samples (**Figure 6A**). Category R (General function prediction) was most abundant (44,110 genes, 9.19%), followed by Category C (Energy production and conversion; 36,165 genes, 7.54%) and Category E (Amino acid transport and metabolism; 35,547 genes, 7.41%) (**Supplementary Table S5**). Analysis of eight core categories (71,731 genes, 19.7% of total) revealed Category M (Cell wall biogenesis) as dominant (20,549 genes, 5.66%), followed by Category T (Signal transduction; 19,559 genes, 5.38%) and Category V (Defense mechanisms; 11,837 genes, 3.26%) (**Figure 6B**). All eight categories exhibited declining abundance trends, with Category T showing the strongest reduction (slope = -264.9), followed by M (slope = -242.0) and V (slope = -150.4) (**Figure 6C**). This widespread functional decline occurred despite generally stable or increasing gene counts in five categories, revealing a disconnect between genetic capacity and functional output (**Figure 6D**). However, five categories exhibited non-significantly increasing gene counts, while two categories showed non-significant decreases. Therefore, an overall relative stability in gene counts rather than abundance.

Correlation analysis between gene count and abundance trends revealed a moderate positive relationship (r = 0.417) (*p = 0.304*). This indicates that while some coordination exists between genetic composition and functional output, substantial independent variation occurs (**Figure 6D**). Quadrant analysis identified divergent patterns in five categories where gene counts increased while functional abundance decreased, particularly in cell wall biogenesis, intracellular trafficking, and secondary metabolism (**Figures 6E, F**). The remaining three categories (V, T, W) showed consonant decreases in both metrics. The temporal trajectory demonstrates clear functional restructuring, with particular significance observed at the YC_1_LD_1 (Year 1-4) sampling point emerging as a critical transition phase, representing both peak total abundance (15,511) and the culmination of a stable functional phase (**Figure 6G**). This period demonstrated strong internal coherence within Cluster 1 (YC_1_XM_1 to YC_1_LD_1) (**Figure 6H**), corresponding with synchronized defense responses in antibiotic resistance patterns (slope = 31.736, R² = 0.825) (**Figure 3B**). Subsequent sampling points revealed dramatic functional restructuring, with YC_1_SD_1 (Year 1-5) showing substantially reduced abundance (2,975) that aligned with the antibiotic resistance collapse phase, followed by functional recovery at YC_1_YJ_1 (**Figure 3**). Correlation analysis revealed near perfect coordination in functional abundance (mean = 0.995) across categories, particularly involving V, T, and Q. Hierarchical clustering identified three functional modules, with Cluster 1 (V, T, Q) showing the strongest internal coordination (mean r = 0.783) (**Supplementary Table S5**).

### 3.7 Community succession reveals structural conservation amid progressive diversity loss

**Figure 7:**
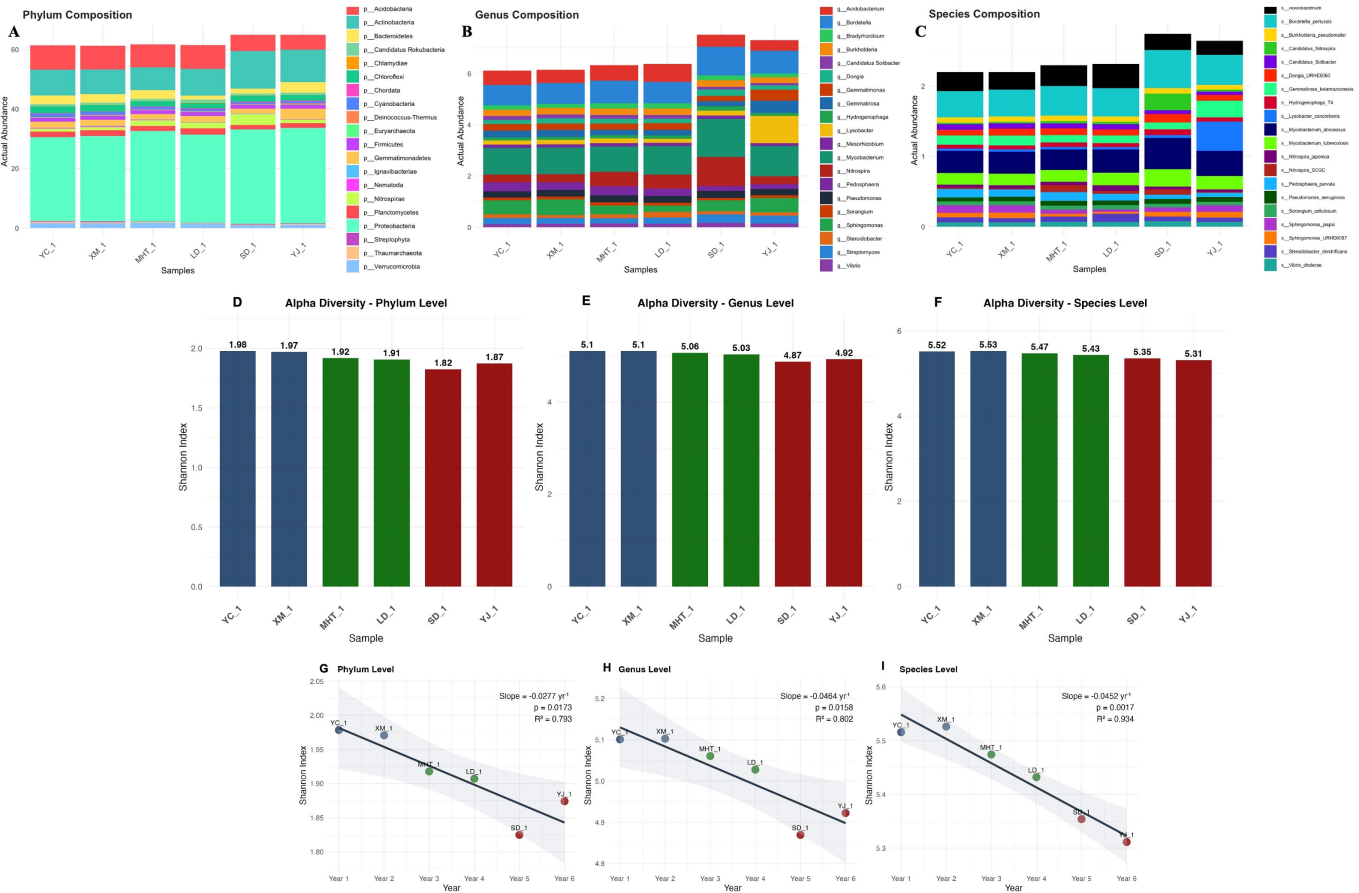
Taxonomic composition across six-year temporal gradient. **(A)** Phylum, **(B)** Genus, **(C)** Species levels. Alpha diversity Bar plots by level, **(D)** Phylum, **(E)** Genus, **(F)** Species levels. Year-group comparisons of Shannon Index **(G)** Phylum, **(H)** Genus, **(I)** Species levels.

Longitudinal analysis of soil microbial assemblages revealed substantial restructuring across the six-year (YC_1 → XM_1 → MHT_1 → LD_1 → SD_1 → YJ_1) monitoring period, demonstrating both structural conservation and progressive diversity erosion. Comprehensive taxonomic profiling identified 1,338 distinct features spanning 66 phyla, 555 genera, and 717 species, revealing a complex hierarchy of successional responses across phylogenetic resolutions. The community maintained a remarkably stable core architecture despite underlying compositional shifts (**Figures 7A–C**). Proteobacteria consistently represented the dominant phylum (28.2-32.1% relative abundance), while Mycobacterium (0.997-1.497) and Bordetella pertussis (0.377-0.542) represented persistent dominant taxa at genus and species levels, respectively, throughout all sampling intervals. This persistent dominance hierarchy suggests strong environmental selection pressure and niche conservatism fundamentally shaping community assembly. Concurrently, systematic erosion of alpha diversity was observed across all taxonomic strata (**Figures 7D–F**). Shannon diversity indices exhibited significant negative successional trends at phylum (slope = -0.0277/yr, *p = 0.0173*), genus (slope = -0.0464/yr, *p = 0.0158*), and species levels (slope = -0.0452/yr, *p = 0.0017*). Year-group comparisons revealed progressive diversity erosion, with maximal diversity in Years 1-2 (Shannon: 1.975 phylum, 5.102 genus, 5.521 species) and lowest in Years 5-6 (1.849, 4.896, 5.332), supported by significant ANOVA results across all levels (*p < 0.05*) (**Figures 7G–I**) (**Supplementary Table S6**).

**Figure 8:**
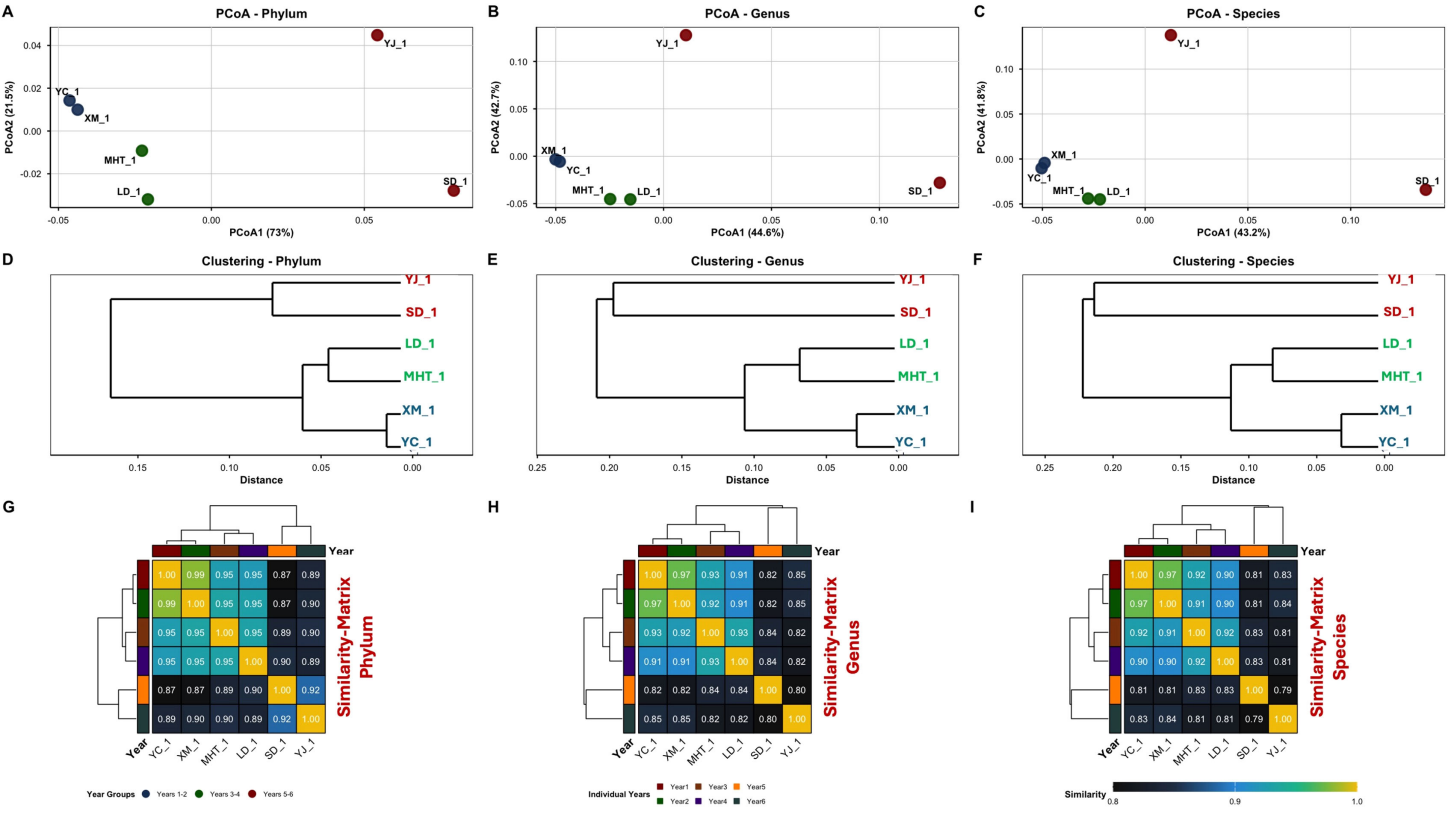
Beta diversity and community structure **(A–C)** PCoA plots, **(D–F)** Hierarchical clustering, **(G–I)** Similarity matrices.

Beta diversity patterns revealed distinct successional clustering of community composition (**Figure 8**). Principal Coordinate Analysis explained substantial variance across taxonomic levels (94.2% phylum, 87.3% genus, 85.0% species), with clear segregation between early (Years 1-4) and late (Years 5-6) temporal clusters (**Figures 8A–C**). Hierarchical clustering consistently resolved two successional metacommunities across all phylogenetic resolutions (**Figures 8D–F**), indicating coordinated community reorganization through time. Cross taxonomic analysis revealed decreasing community similarity with increasing phylogenetic resolution, from 91.9% mean similarity at phylum level to 85.8% at species level (**Figures 8G–I**). Temporal distance decay relationships revealed systematic community turnover, with the phylum level exhibiting the strongest successional signal (slope = +0.0164 yr, R² = 0.346, *p = 0.021*), equivalent to 1.6% annual community dissimilarity (**Supplementary Table S6**). Temporal gradients emerged were further examined through within and between year group dissimilarity metrics (**Supplementary Figure S3**). Notably, observed richness revealed distinct successional dynamics across taxonomic levels (**Supplementary Figure S4**). While phylum-level richness remained relatively stable (45–47 phyla per sample), species-level richness showed subtle declines from early (717 species) to late successional stages (715 species). This divergence between maintained richness and declining diversity indices indicates progressive unevenness in species abundance distributions, where dominant taxa consolidating their proportional representation while rare species persist at diminished abundances.

**Figure 9:**
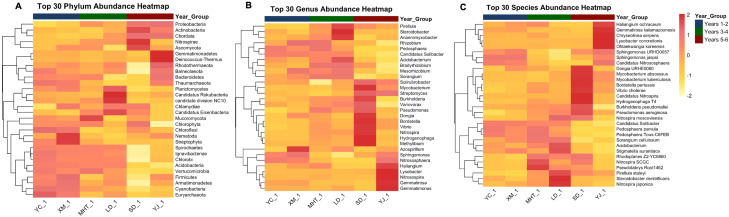
Heatmap visualizations of abundant 30 taxa **(A)** Phylum, **(B)** Genus, **(C)** Species levels.

**Figure 10:**
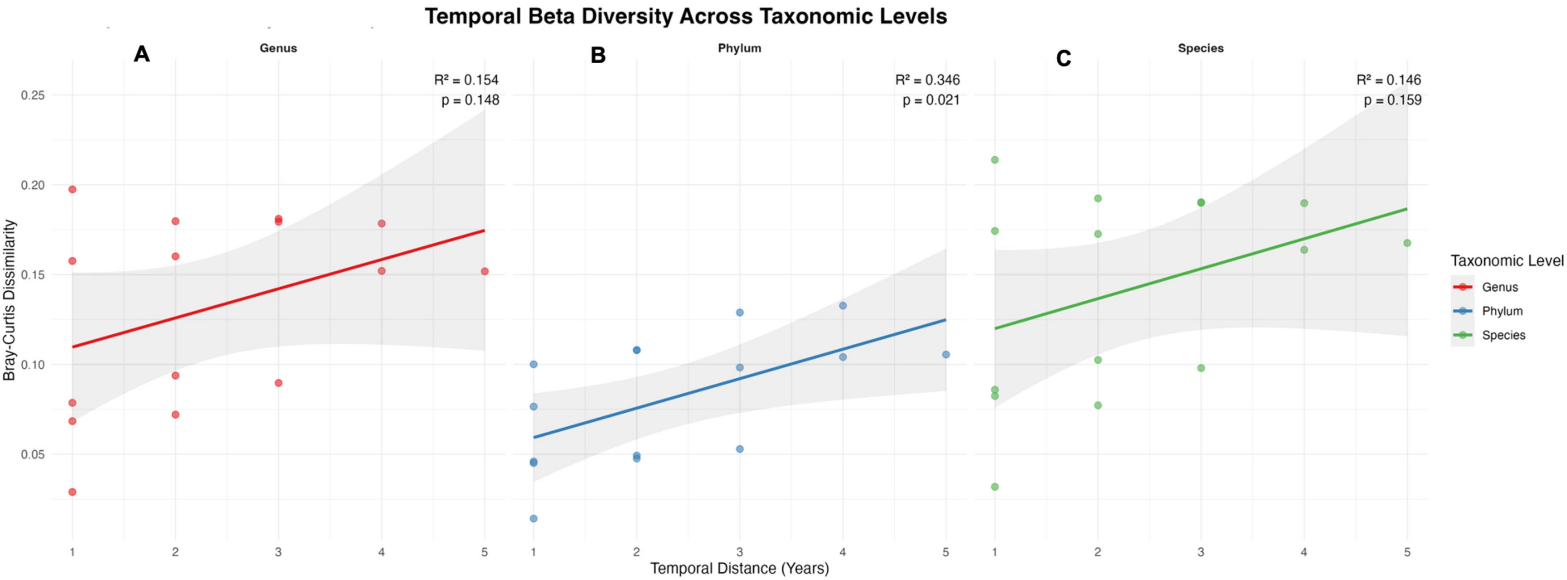
Temporal beta diversity pattern **(A)** Genus **(B)** Phylum **(C)** Species.

Heatmap visualization of abundance pattern revealed this dynamic explicitly (**Figures 9A–C**), with the presence of top 30 taxa at each level across all time points alongside substantial abundance fluctuations. Actinobacteria emerged as the most dynamic phylum (SD = 1.89), while *Lysobacter* showed greatest genus-level variability (SD = 0.36), indicating differential environmental responsiveness among dominant taxonomic groups. Temporal analysis further revealed non-linear community dynamics, with within group dissimilarity increasing progressively across the sampling period (**Figures 10A–C**). At the phylum level, dissimilarity within year groups increased from 0.014 (Years 1-2) to 0.077 (Years 5-6), indicating accelerating community change in later successional stages. This pattern was substantially amplified at finer taxonomic resolutions, with genus and species levels showing substantially higher within-group dissimilarity in later years (0.197 and 0.214, respectively). The integration of these conserved dominance hierarchies, declining diversity, and increasing temporal turnover supports a successional model where environmental filtering primarily restructuring community evenness rather than species presence-absence relationships. This suggests that functional redundancy within the rare biosphere buffers ecological resilience against compositional changes, while core taxa maintain ecosystem functions through temporal environmental variation. The maintenance of high species richness (714–717 species) alongside declining diversity indices and increasing temporal turnover indicates a successional trajectory where competitive exclusion and environmental filtering progressively reshape community structure without eliminating taxonomic diversity, highlighting the complex resilience mechanism operating within soil microbial communities.

## 4 Discussion

The management of soil borne plant pathogens represents one of the most persistent challenges in modern agriculture, with conventional control strategies often proving inadequate against resilient pathogens like *P. nicotianae* (Erwin and Ribeiro, 1996; Shew, 1983). Our six-year longitudinal study revealed that continuous disease pressure induces profound, non-equilibrium restructuring across the soil microbiome’s functional domains, challenging static pathosystems models. The significance of our findings lies in demonstrating that soil microbial communities do not exist in stable equilibrium but undergo continuous restructuring in response to persistent pathogen pressure particularly the soil borne pathogens like *Phytophthora* and the progressive transcriptional landscape. We observed substantial annual transcriptional restructuring, with differentially expressed genes progressively increasing, culminating in 1,006 highly significant genes in the six-year comparison. This cumulative transcriptional reprogramming suggests continuous molecular adaptation under persistent pathogen pressure, indicating that agricultural management must account for temporal dynamics rather than snapshot assessments (Shade et al., 2012), therefore understanding of soil pathosystems requires longitudinal observation across multiple years. The substantial bidirectional regulation observed across all comparisons reveals complex regulatory networks that enable microbial communities to maintain functional plasticity while responding to changing environmental conditions.

Functional profiling analysis exhibited systematic metabolic adaptation, with a clear trend toward enrichment of core biosynthetic processes and microbial coordination. The progressive increase in KOs abundance from 46.8% in early years (1-3) to 66.7% in the cumulative (3-6) comparison suggests ongoing microbial community optimization, consistent with ecological succession patterns observed in long-term microbial studies (Shade et al., 2012). The substantial increases in amino acid biosynthesis, carbon metabolism, and ABC transporters indicate enhanced nutrient processing and transport capabilities, like metabolic enrichments where microbial communities often show predictable functional changes in response to environmental pressures like nutrient conditions (Fierer et al., 2012). Particularly, coordinated enhancement of quorum sensing components (11%) and ribosome abundance (13%), suggesting that microbial communities under stress invest in both signal transduction and protein synthesis capacity that reinforced microbial communication networks which regulate collective behaviors including biofilm formation (Nemergut et al., 2013; Waters and Bassler, 2005). Decreased pathways, particularly the 39.3% reduction in glycan degradation, may reflect optimized resource allocation, consistent with functional trade-offs in adapting communities (Langenheder and Székely, 2011). The Year 4–5 transition emerged as a critical adaptation threshold, showing the strongest positive shifts in functional potential with predominance of increasing pathways (317 vs 70 decreasing (Konopka et al., 2015; Goldfarb et al., 2011) where microbial communities prioritize core functions essential for survival and coordination.

Resistome profiling revealed dramatic ecological succession: coordinated defense in Year 2–3 aligned with the “cry for help” hypothesis (Yuan et al., 2018), comprehensive collapse in Year 3–4 involving critical multidrug efflux system failure (Li et al., 2015b), and targeted recovery with bleomycin resistance adaptation to oxidative stress (Van Acker and Coenye, 2017). The system remained in continuous flux rather than reaching equilibrium. Virulence factor analysis revealed evolutionary trade-offs rather than pathogenicity escalation. Flagella dominance suggests enhanced motility as a primary adaptive strategy (de Weert et al., 2002; Lugtenberg and Kamilova, 2009), while balanced regulation (48.6% increasing vs 51.4% decreasing) challenges arms race dynamics, reflecting metabolic optimization (MacLean and Gudelj, 2006). Strategic shifts toward contact dependent pathogenesis and energy conservation were evident, with secretion system decreases likely reflecting substantial energetic costs (Preston, 2007). Persistence analysis identified core virulence elements, with ABC transporter specialization suggesting adaptive responses to monoculture induced nutrient gradients (Solomon et al., 2003; Büttner and Bonas, 2010). The *Phytophthora*-focused analysis reveals distinct pathogenic strategies between oomycete and bacterial communities within the same agricultural ecosystem. The retention of adhesion mechanisms and specialized secretion systems as primary *Phytophthora* virulence factors underscores the evolutionary divergence in pathogenesis mechanisms between these pathogen groups. Oomycetes, unlike many bacteria, depend heavily on these mechanisms for initial host attachment and penetration, a distinction well documented in comparative pathology (Tyler, 2002). The variable regulation patterns within flagella systems with general flagella increasing while specific polar flagella subtypes decrease suggests functional specialization and potential niche partitioning among pathogen populations. This diversification represents an adaptive response to heterogeneous soil microenvironments, allowing different pathogen lineages to exploit specific ecological niches. Such population-level specialization has been documented in other soil-borne pathogen systems and contributes to long-term persistence in agricultural soils (Mazzola, 2004). Collectively, these findings challenge the conventional view that continuous monoculture inevitably leads to virulence escalation. Instead, we observe a more nuanced adaptation characterized by strategic optimization of virulence investments. This pattern supports emerging understanding that pathogen evolution in agricultural systems involves complex trade-offs between virulence, transmission, and survival, rather than simple directional selection for increased aggressiveness (Sacristán and García-Arenal, 2008; Howlett et al., 2015). The balanced regulation of Stress Response mechanisms (49.1% upregulated) despite overall decreasing trends in other virulence categories suggests that pathogens maintain capacity to respond to environmental challenges while optimizing energy expenditure. This strategic preservation of stress response capabilities has been observed in other microbial systems adapting to predictable environmental stresses (Ferenci, 2016).

CAZy analysis revealed class-level stability (GT: 38.8%, CE: 29.6%) with family-level plasticity. GT41 and GT4 persistence reflects essential glycosylation roles (Cantarel et al., 2009; Yip and Withers, 2006), while extreme variability in GH102 (peptidoglycan lytic transglycosylases) (Blackburn and Clarke, 2001), CBM4 (multi-substrate binding) (Cantarel et al., 2009; Boraston et al., 2004), and PL15 (alginate lyase activity) (Cantarel et al., 2009; Yip and Withers, 2006) indicates context dependent adaptation. Network analysis revealed coordinated modular operation (404 edges, density: 0.167), enabling functional reliability with adaptive flexibility (Blackburn and Clarke, 2001; Boraston et al., 2004). COG profiling revealed profound functional restructuring and genetic functional decoupling. Widespread functional abundance declines across all eight core categories despite stable/increasing gene counts suggest systemic metabolic downregulation (Blazewicz et al., 2013; Jansson and Hofmockel, 2020). Critical transitions at YC_1_LD_1 (functional peak) and YC_1_SD_1 (vulnerability window) resemble stress response patterns (Shade et al., 2012), while near perfect functional coordination (mean r = 0.995) indicates synchronized community-level responses This decoupling highlights that genomic inventories may overestimate operational capabilities in stressed communities (Jansson and Hofmockel, 2020; Fierer et al., 2021).

We further observed that the diversity analysis revealed a nuanced successional model where environmental changes primarily restructure community evenness rather than species presence-absence relationships. The maintenance of high species richness alongside declining diversity indices and increasing temporal turnover indicates a trajectory where competitive exclusion and environmental filtering progressively reshape community structure without eliminating taxonomic diversity. This suggests that functional redundancy within the rare biosphere provides ecological resilience against compositional changes, while core taxa maintain essential ecosystem functions through temporal environmental variation. Moreover, broader context of soil health and agricultural sustainability is crucial for interpreting our results. The degradation of soil microbial diversity and fertility through intensive monocropping and agrochemical use, as reported by Geisseler and Scow (2014), Wu et al. (2008), and Xuan et al. (2012), creates conditions where pathosystems can become increasingly difficult to manage. Our findings demonstrating declining alpha diversity across all taxonomic levels support these concerns, while also revealing the remarkable resilience mechanisms that soil communities employ to maintain function. The complex interactions in the rhizosphere, emphasized by Bais et al (Bais et al., 2006). and Raaijmakers et al (Raaijmakers et al., 2009). as a critical hotspot for microbial activity, appear to be fundamentally reshaped by long-term monoculture and pathogen pressure.

Collectively, our study showed that long-term *Phytophthora* pressure drives the soil microbiome toward a state of heightened and coordinated defensiveness, with profound implications for soil health and ecosystem functioning. A microbiome primed for constant defense may trade-off other key soil functions as microbial resources are diverted to maintenance and survival (Jechalke et al., 2014). Furthermore, the enrichment of resistance mechanisms to clinically important drug classes in an agricultural setting raises concerns about environmental antibiotic resistance and potential horizontal gene transfer to human pathogens (Perry and Wright, 2013). The most significant implication of our research revealed that soil microbial communities maintain functional resilience through temporal adaptation rather than static resistance. The maintenance of high taxonomic diversity alongside declining evenness and increasing functional specialization reveals a sophisticated ecological strategy where functional redundancy provides buffer capacity while core taxa maintain essential functions (Allison and Martiny, 2008). The continuous adaptation cycle observed throughout our six-year study underscores that plant pathogens exert lasting impacts on soil microbial communities, driving dynamic restructuring that represents a tangible, long-term alteration of the soil ecosystem with significant consequences for sustainable agriculture as resilience may be more important than resistance in maintaining ecosystem functions under stress (Chen et al., 2020; Dubey et al., 2019).

## 5 Conclusions

In conclusion, our six-year longitudinal analysis reveals that agricultural pathosystems are characterized by continuous adaptation across multiple biological levels. Rather than reaching stable equilibrium, soil microbial communities undergo progressive restructuring that involves transcriptional reprogramming, metabolic optimization, and functional specialization. These findings challenge static models of soil ecosystems and highlight the need for temporal perspectives in agricultural management. By revealing the sophisticated adaptation strategies employed by both pathogens and beneficial microbes, our research provides a foundation for developing dynamic, ecologically informed approaches to sustainable disease management that account for the complex temporal dynamics of agricultural ecosystems.

## Data Availability

The datasets presented in this study can be found in the online repository at: https://github.com/UmerBasu/Phytopthora_Metagenome_analysis_results_Basu-et-al.-2025.

## Supplementary material

Supplementary Figure 1 Density plots showing log2 FC distributions for six sequential year comparisons: Year 1 vs 2, Year 2 vs 3, Year 3 vs 4, Year 4 vs 5, Year 5 vs 6, and Year 1 vs 6. Dashed red line indicates no change (log2FC = 0).

Supplementary Figure 2 Class-specific co-occurrence heatmaps revealing distinct temporal patterns within each enzyme category: **(A)** Carbohydrate-Binding Modules (CBM), **(B)** Glycosyl Transferases (GT), **(C)** Carbohydrate Esterases (CE), **(D)** Polysaccharide Lyases (PL), **(E)** Glycoside Hydrolases (GH).

Supplementary Figure 3 Temporal comparisons: **(A)** Year group dissimilarities: Within-group (Years 1-2: 0.014-0.032, Years 3-4: 0.046-0.082, Years 5-6: 0.077-0.214); Between group (Years 1–2 vs 3-4: 0.049-0.091, Years 3–4 vs 5-6: 0.104-0.182). **(B)** Extreme comparisons: Largest gap 5yr (0.105-0.168), Most similar distant 3yr (0.053-0.098), Least similar close 1-2yr (0.108-0.214). Bray-Curtis dissimilarity 0–1 scale.

Supplementary Figure 4 Richness trends: Phylum (slope=-0.200, p=0.427), Genus (slope=-1.800, p=0.235), Species (slope=-0.400, p=0.641). Range: Phylum 45-47, Genus 545-555, Species 714-717.

Supplementary Table 1 KEGG pathway abundance changes between early and late sampling periods.

Supplementary Table 2 Summary statistics of antibiotic resistance gene dynamics across year comparisons.

Supplementary Table 3 Virulence factor distribution and directional changes.

Supplementary Table 4 Comprehensive CAZy class and family statistics across six-year temporal analysis.

Supplementary Table 5 Comprehensive COG functional category.

Supplementary Table 6 Microbial diversity metrics across taxonomic levels.
