# Supplementary figures and images for: Longitudinal metagenomics reveals continuous restructuring of soil pathobiome under persistent Phytophthora pressure
Source: Front Plant Sci. 2026 Feb 2;16:1749879. doi: 10.3389/fpls.2025.1749879 (PMC12907438; doi:10.3389/fpls.2025.1749879)

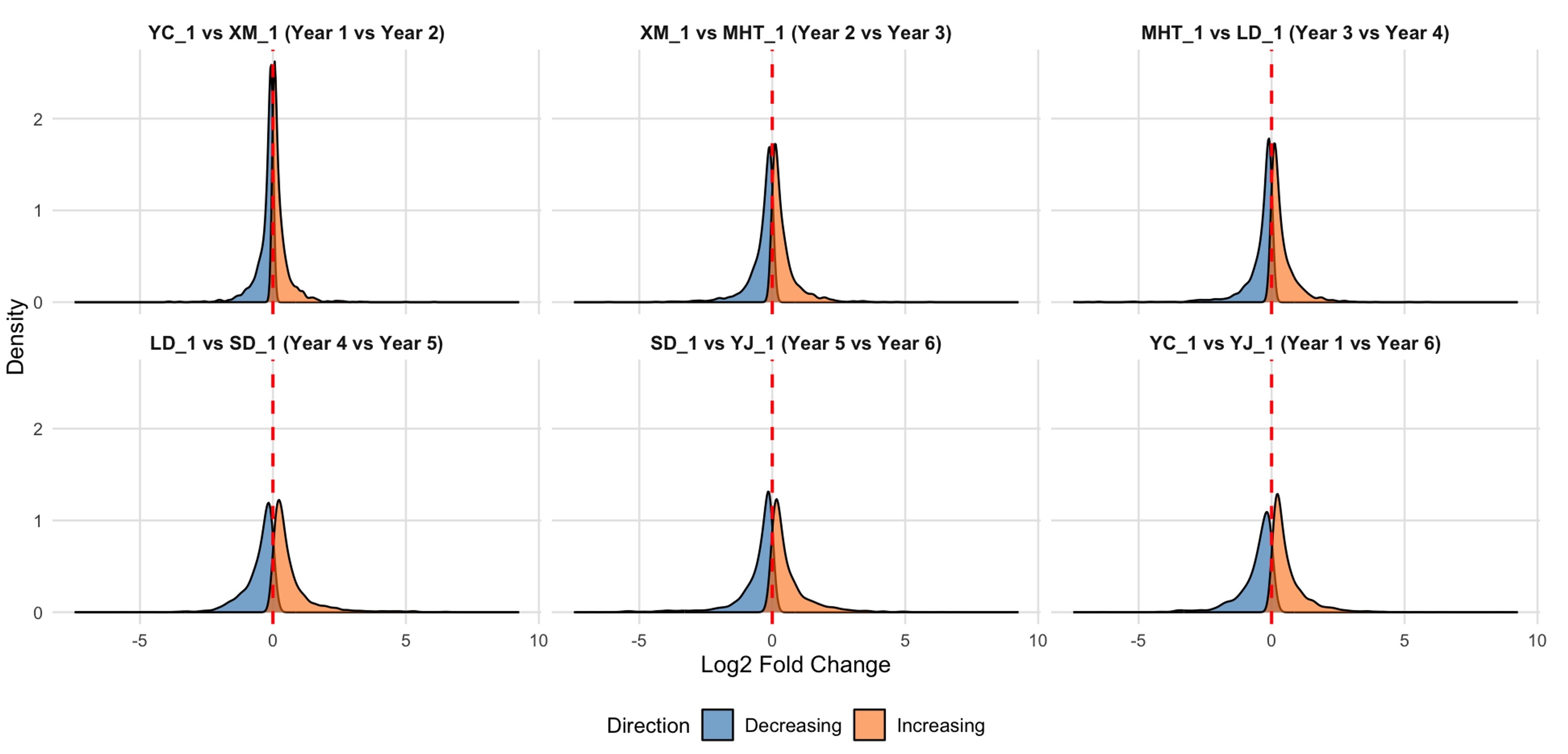

Supplement: Supplementary Figure 1 — Density plots showing log2 FC distributions for six sequential year comparisons: Year 1 vs 2, Year 2 vs 3, Year 3 vs 4, Year 4 vs 5, Year 5 vs 6, and Year 1 vs 6. Dashed red line indicates no change (log2FC = 0). [file Image1.jpeg]

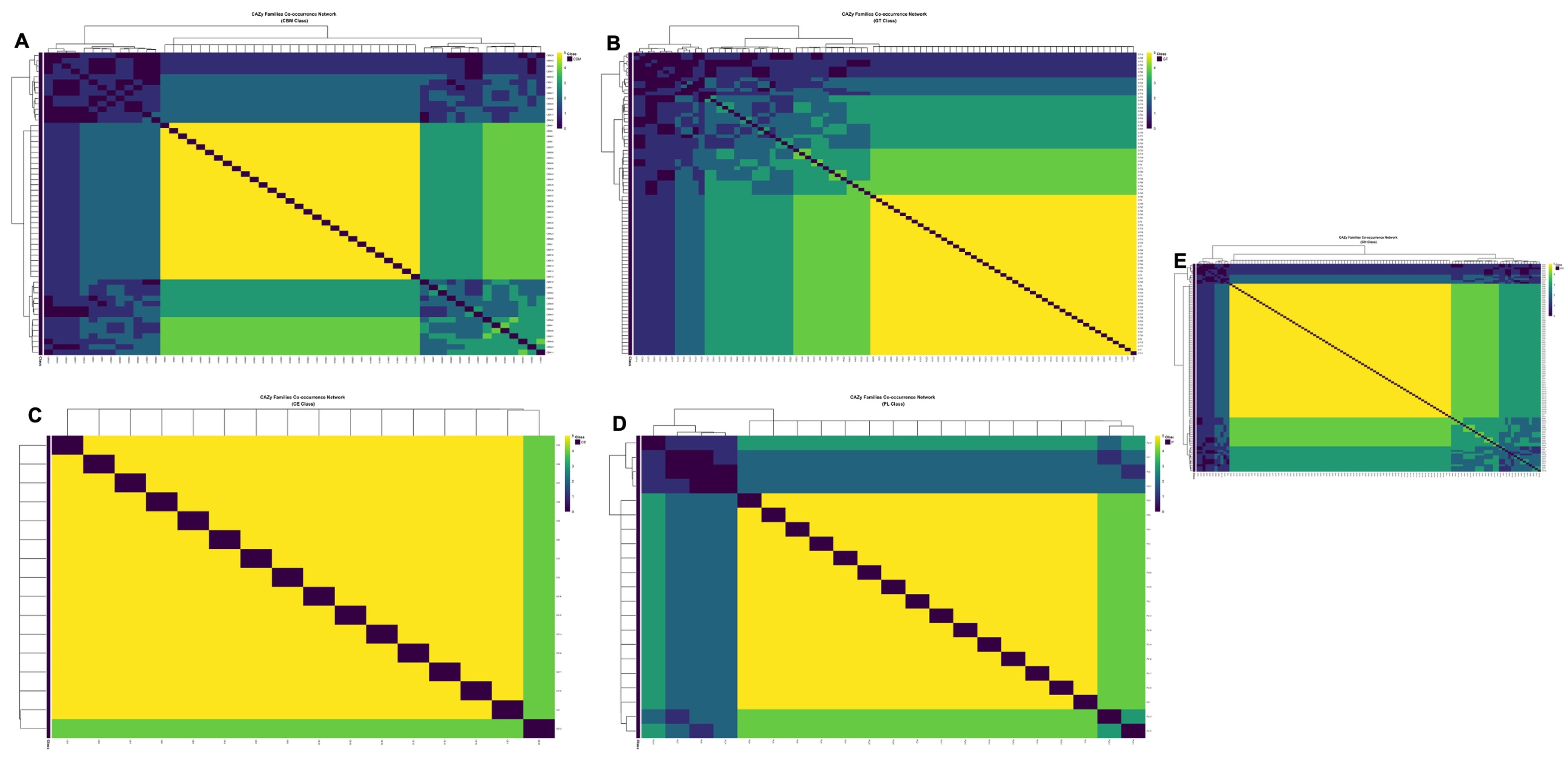

Supplement: Supplementary Figure 2 — Class-specific co-occurrence heatmaps revealing distinct temporal patterns within each enzyme category: (A) Carbohydrate-Binding Modules (CBM), (B) Glycosyl Transferases (GT), (C) Carbohydrate Esterases (CE), (D) Polysaccharide Lyases (PL), (E) Glycoside Hydrolases (GH). [file Image2.jpeg]

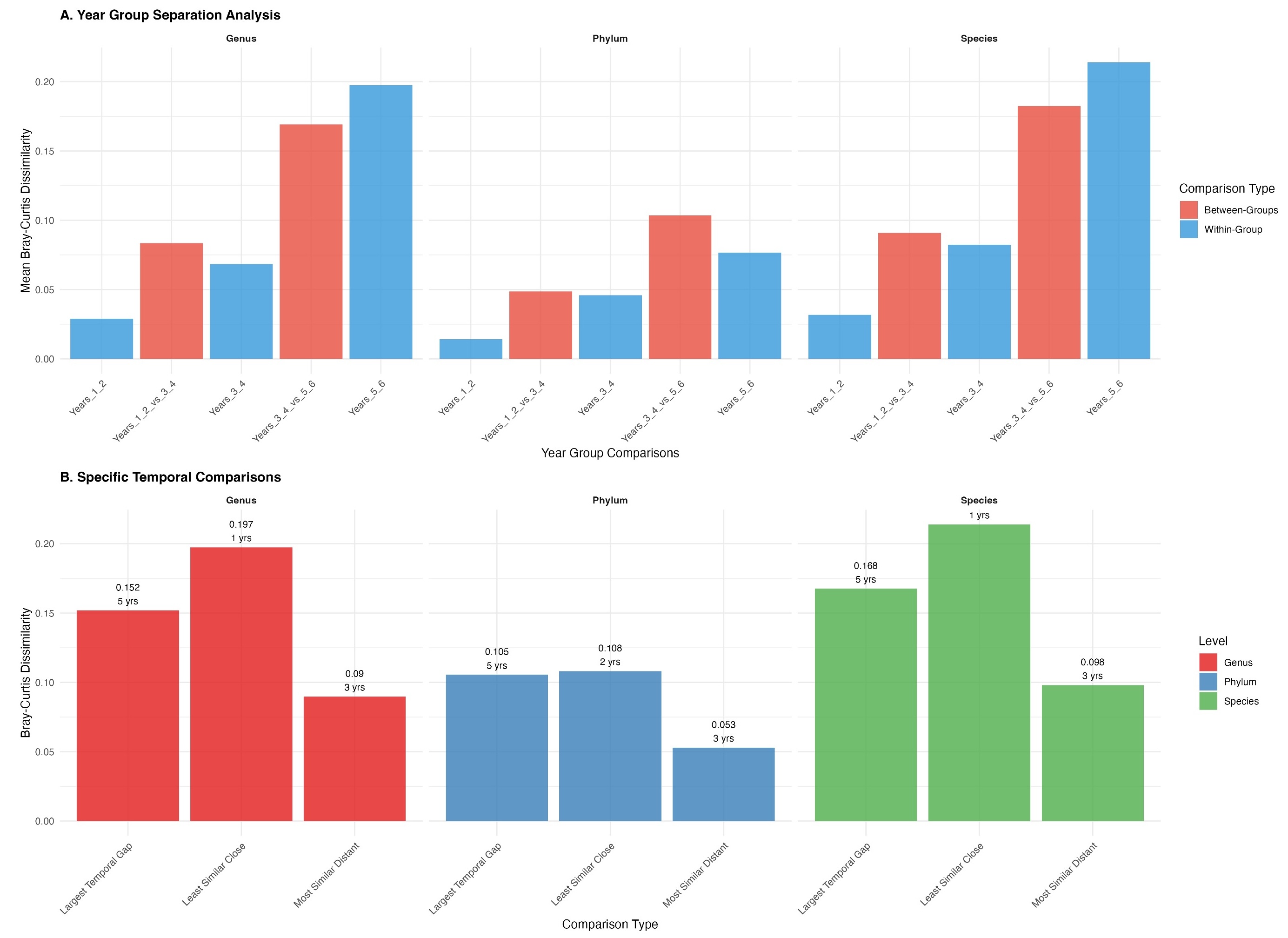

Supplement: Supplementary Figure 3 — Temporal comparisons: (A) Year group dissimilarities: Within-group (Years 1-2: 0.014-0.032, Years 3-4: 0.046-0.082, Years 5-6: 0.077-0.214); Between group (Years 1–2 vs 3-4: 0.049-0.091, Years 3–4 vs 5-6: 0.104-0.182). (B) Extreme comparisons: Largest gap 5yr (0.105-0.168), Most similar distant 3yr (0.053-0.098), Least similar close 1-2yr (0.108-0.214). Bray-Curtis dissimilarity 0–1 scale. [file Image3.jpeg]

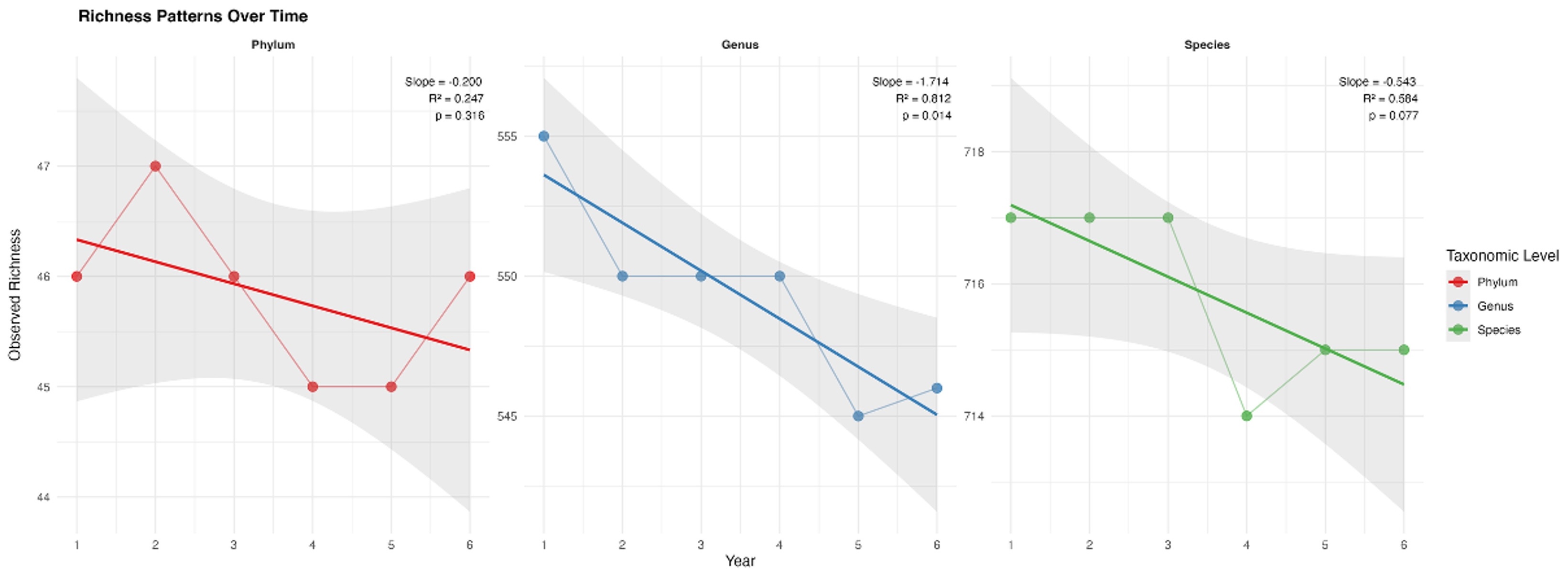

Supplement: Supplementary Figure 4 — Richness trends: Phylum (slope=-0.200, p=0.427), Genus (slope=-1.800, p=0.235), Species (slope=-0.400, p=0.641). Range: Phylum 45-47, Genus 545-555, Species 714-717. [file Image4.jpeg]
